# Associations of plasma metabolites with protein biomarkers linked to Alzheimer’s disease pathology in the Rotterdam Study

**DOI:** 10.1186/s13195-026-02108-2

**Published:** 2026-06-10

**Authors:** Midas M. Kuilman, Shahzad Ahmad, Amos C. Pomp, Frank J. Wolters, Rima Kaddurah-Daouk, M. Arfan Ikram, Mohsen Ghanbari

**Affiliations:** 1 Department of Epidemiology, Erasmus MC University Medical Center Rotterdam, Dr. Molewaterplein 40, Rotterdam, 3015 GD the Netherlands; 2 Sidra Medicine, Doha, Qatar; 3 Department of Psychiatry and Behavioral Sciences, Duke University, Durham, NC USA; 4 Duke Institute of Brain Sciences, Duke University, Durham, NC USA; 5 Department of Medicine, Duke University, Durham, NC USA

**Keywords:** Alzheimer's Disease, Metabolomics, Protein Biomarkers, Triglyceride-Rich Lipoproteins, APOE Genotype

## Abstract

**Background:**

Alzheimer’s disease (AD) is a multifactorial neurodegenerative disorder characterized by amyloid-β (Aβ) and tau pathology, neuroaxonal damage, and neuroinflammation. While blood biomarkers, such as tau, neurofilament light chain (NfL), and Aβ isoforms, reflect AD pathology, the systemic metabolic alterations contributing to the disease remain poorly defined. This study aims to explore associations between plasma metabolites and key protein biomarkers linked to AD pathology in a large, population-based cohort.

**Methods:**

Plasma levels of Aβ40, Aβ42, total-tau (t-tau), and NfL were measured using the highly sensitive Simoa NF-light and N3PA assays (Quanterix platform) among over 3,000 participants from the Rotterdam Study. Plasma metabolites were quantified using the Nightingale NMR-based (*n* = 2,871) and Metabolon MS-based (*n* = 1,491) platforms. Multivariable linear regression models, adjusted for demographic, lifestyle, and genetic factors, were applied. Analyses were stratified by sex and APOE genotype. Sensitivity analyses excluded participants with dementia, stroke, or impaired kidney function.

**Results:**

Triglyceride-rich lipoproteins across VLDL, LDL, and HDL subclasses were positively associated with both Aβ isoforms. Among them, Triglyceride-rich lipoproteins in small VLDL showed the strongest association with Aβ40 (β = 0.168, FDR = 4.362e-16). Conversely, HDL cholesterol fractions showed inverse associations with Aβ. GlycA (β > 0.097, FDR < 2.092e-05) and creatinine (β > 0.253, FDR < 3.371e-24) were positively associated with all Alzheimer’s disease biomarkers, while albumin was inversely related to tau (β = -0.094, FDR = 1.924e-04) and NfL (β = -0.079, FDR = 4.210e-05). On the Metabolon platform, S-adenosylhomocysteine (β > 0.206, FDR < 1.303e-09) was positively associated with all biomarkers, while uridine and 2′-deoxyuridine showed inverse associations with Aβ40, t-tau, and NfL. Stratified analyses indicated stronger GlycA-tau associations in APOE ε2 carriers and sex-specific effects for amino acids and ApoA1.

**Conclusions:**

Our findings highlight metabolites involved in lipid transport, inflammation, amino acid metabolism, and methylation as being linked to AD-related protein biomarkers. These results provide novel insights into systemic metabolic pathways underlying AD and suggest that certain metabolites may serve as potential biomarkers for early detection and risk stratification in AD.

**Supplementary Information:**

The online version contains supplementary material available at https://doi.org/10.1186/s13195-026-02108-2.

## Background

Alzheimer’s disease is the most common neurodegenerative disorder and the leading cause of dementia worldwide [1]. Although our understanding of Alzheimer’s disease pathology has advanced, the underlying molecular mechanisms remain incompletely understood, and effective disease-modifying therapies are still lacking. Alzheimer’s disease pathology is primarily defined by amyloid-β (Aβ) plaques and neurofibrillary tangles [2], with synaptic dysfunction and neuroinflammation further contributing to disease progression [3].

In blood, the most widely studied biomarkers for Alzheimer’s disease include tau, neurofilament light chain (NfL), and the Aβ isoforms Aβ40 and Aβ42. Tau reflects neurofibrillary tangle pathology and is closely linked to microtubule destabilization, axonal transport dysfunction, and progressive neuronal injury, representing a key downstream process of Alzheimer’s disease neurodegeneration. NfL serves as a sensitive but non-specific marker of axonal damage and neurodegeneration across neurological conditions. Aβ40 and Aβ42 capture amyloid production and aggregation dynamics related to plaque formation, with the Aβ42/40 ratio commonly used to improve robustness and reduce inter-individual variability [4, 5]. Beyond their clinical value for diagnosis and disease staging, these blood-based biomarkers could serve as proxies to investigate systemic processes that contribute to Alzheimer’s disease pathology. However, interpretation of these biomarkers in plasma requires caution. Plasma tau levels show weaker specificity for Alzheimer’s disease compared with cerebrospinal fluid or imaging markers, NfL reflects general neurodegeneration rather than disease-specific processes, and circulating Aβ levels are influenced by both central and peripheral sources as well as assay-related variability. Despite these limitations, blood-based biomarkers provide a minimally invasive and scalable approach to capture key aspects of Alzheimer’s disease pathology and are increasingly used in large population-based studies. Recently, the U.S. Food and Drug Administration (FDA) approved a blood-based diagnostic test that measures phosphorylated-tau and Aβ42, marking a major step toward less invasive Alzheimer’s disease diagnosis and underscoring the growing role of fluid biomarkers in both research and clinical practice [6].

Plasma levels of these biomarkers reflect both central and peripheral processes and may not directly map onto brain pathology, but still provide valuable insight into disease-related biology. While current Alzheimer’s disease biomarkers primarily capture amyloid and tau pathology, early metabolic alterations affecting lipid homeostasis, energy metabolism, neurotransmission, and oxidative stress, remain less well characterized, despite emerging years before clinical symptoms [7]. By linking plasma metabolite profiles with blood-based Alzheimer’s disease biomarkers such as NfL, tau, and Aβ proteins, we can gain insight into underlying neurodegenerative pathways, identify novel mechanisms, and improve risk stratification. High-throughput metabolomics platforms, such as Nightingale and Metabolon, enable comprehensive metabolomic profiling, which is increasingly used to study the relationship between metabolic dysfunction and dementia risk. Metabolic syndrome, characterized by a cluster of conditions like dyslipidemia, insulin resistance, and hypertension, is known to increase dementia risk [8]. We hypothesize that lipid-related metabolites are associated with amyloid-related biomarkers (Aβ40 and Aβ42), inflammatory and immune-related pathways with markers of neurodegeneration (NfL and tau), and amino acid and one-carbon metabolism with broader neuronal and metabolic dysfunction reflected across these biomarkers. The metabolomics data from these platforms may help us better understand how these conditions at the molecular level contribute to neurodegeneration.

Given the central role of lipid metabolism, attention has also focused on the apolipoprotein E (APOE) gene, with the ε4 allele representing the strongest common genetic risk factor for late-onset Alzheimer’s disease, influencing amyloid clearance, lipid metabolism, and neuroinflammation [9]. Likewise, sex differences are well documented in Alzheimer’s disease risk, biomarker profiles, and cognitive decline trajectories [10]. These observations suggest that associations between systemic metabolites and Alzheimer’s disease biomarkers may differ by APOE genotype or sex. However, only a few metabolomics studies have systematically examined a broad range of plasma metabolites in relation to multiple blood-based Alzheimer’s disease biomarkers while accounting for heterogeneity by APOE genotype and sex, and most rely on subset of targeted metabolites. To fill these gaps, we investigated a wide-range of plasma metabolites from two established metabolomics platforms, the Nightingale and the Metabolon, in combination with four well-known protein biomarkers related to hallmarks of Alzheimer’s disease (NfL, t-tau, Aβ40, and Aβ42), within the population-based Rotterdam Study [11]. Our aim was to characterize systemic metabolic signatures associated with Alzheimer’s disease pathology and to test whether these associations differ by APOE genotype and sex, providing new insights into the metabolic underpinnings of Alzheimer’s disease.

## Methods

### Study design and population

This study is embedded within the Rotterdam Study [11], a large, ongoing, prospective population-based cohort initiated in 1990 in the Ommoord district of Rotterdam, the Netherlands. The study was set up to investigate the incidence, risk factors, and progression of chronic and age-related diseases in the general population. All inhabitants aged 55 years and older from the Ommoord were invited to participate. The initial cohort (RS-I) included 7,983 participants enrolled between 1989 and 1993. Three additional cohorts (RS-II, III and IV) were subsequently recruited. As of February 2024, the Rotterdam Study contains 17,931 participants aged 40 years and older, with in-person examinations every 3 to 5 years, in addition to continuous surveillance through electronic linkage with healthcare providers.

The Rotterdam Study is approved by the Erasmus MC Medical Ethics Committee (MEC 02.1015) and the Dutch Ministry of Health (license 1071272-159521-PG). It follows the Declaration of Helsinki principles and is registered with the Erasmus MC Data Protection Officer (EMC1712001). The study’s persistent identifier is https://ror.org/02ac58f22. All participants gave written informed consent to participate and allow access to their medical data [11].

For the current analyses, we included 2,958 participants from the fourth visit of RS-I (RS-I-4) with available measurements for both plasma Alzheimer’s disease protein biomarkers and metabolomic data derived from the Nightingale and Metabolon platforms. Metabolite measurements were conducted in participants with available informed consent, plasma samples, and covariate data using the Nightingale platform, with a random subsampling for the Metabolon platform. Blood samples were collected between 8:00 and 10:30 AM following an overnight fast. Blood was drawn into EDTA tubes, centrifuged, aliquoted, and stored at -80 °C until analysis.

### Nightingale health metabolomics platform

Metabolites were quantified from fasting EDTA plasma samples using a high-throughput proton nuclear magnetic resonance (NMR) metabolomics platform developed by Nightingale Health Ltd. (Helsinki, Finland). This metabolite profiling was done as part of the 4th Rainbow Project of BioBanking for Medical Research Infrastructure of the Netherlands (BBMRI-NL) (https://www.bbmri.nl/omicsmetabolomics/). All samples were stored at -80 °C, which ensured biological stability. This targeted platform provides absolute quantification of a broad range of circulating metabolites, including lipids, lipoprotein subclasses, fatty acids, amino acids, ketone bodies, and markers of inflammation. A total of 230 metabolite measures were available, of which 167 absolute concentrations (excluding metabolite ratios and percentages) were selected for analysis in this study. Metabolites with zero concentrations were treated as missing and samples with > 40% missingness were excluded. Metabolites were log_2_ transformed to approximate normality, and remaining missing values were imputed using k-nearest neighbors (kNN). Sample outliers were removed using the local outlier factor method, and extreme metabolite values were subsequently imputed with kNN. The metabolite measurements were then scaled to standard deviation units (mean 0, SD 1) to enable the comparison of results for measures with different units.

### Metabolon platform

Untargeted metabolomics profiling was generated by the Alzheimer Disease Metabolomics Consortium using the Metabolon HD4 platform, based on ultra-high-performance liquid chromatography coupled with tandem mass spectrometry (UPLC-MS/MS). This approach enables a broad, discovery-oriented capture of endogenous and exogenous metabolites. A total of 1,387 named and unnamed metabolites across multiple chemical classes, including lipids, amino acids, xenobiotics, nucleotides, cofactors and vitamins, peptides, carbohydrates, and energy-related compounds were quantified based on their relative abundances, calculated using the area under the curve of detected peaks. To correct for inter-day instrument variation, a block correction was applied by scaling the median value of each metabolite per run-day to 1.00, with all other values adjusted proportionally. Before performing the imputation, we excluded 14 participants with missingness greater than 5 times the standard deviation of the mean missingness in all participants, resulting in a sample size of 1,082. Metabolites with more than 70% missing values were excluded. We further excluded metabolites with a coefficient of variance greater than 30%, leaving 1111 metabolites after the quality control. For this study, we included 991 metabolites with ≤ 30% missingness. After log_2_ transformation, missing values were imputed using the kNN method, which has previously been shown to perform robustly in metabolomics data. A total of 868 named metabolites spanning multiple biochemical pathways were retained for analysis.

### Alzheimer’s disease associated protein biomarkers

Plasma concentrations of neurofilament light chain (NfL), t-tau, amyloid-beta 40 (Aβ40), and amyloid-beta 42 (Aβ42), were measured using the Single Molecule Array (Simoa) HD-1 analyzer (Quanterix). NfL was quantified using the Simoa NF-light^®^ Advantage Kit, while t-tau, Aβ40, and Aβ42 were measured using the Simoa Human Neurology 3-Plex A (N3PA) assay. All measurements were performed in duplicate, and each assay plate included two internal quality control samples per analyte. Measurements were excluded from analysis if duplicates were missing, if the coefficient of variation exceeded 20%, or if quality control values fell outside acceptable ranges. Values were log_2_-transformed and standardized before statistical analysis.

### Statistical analysis

Prior to modeling, metabolite values from both platforms were log_2_-transformed. The log-transformed values were then standardized to units of standard deviation. We ran linear models for both Nightingale, Metabolon with identical covariates, repeated in sex and APOE strata, and controlled FDR at 5%. We used multivariable linear regression to examine associations between individual metabolites and four Alzheimer’s disease biomarkers (Aβ40, Aβ42, t-tau, and NfL). All models were adjusted for age, sex, body mass index (BMI), education level, smoking status, alcohol consumption, use of lipid-lowering medication, estimated glomerular filtration rate (eGFR), and APOE carrier status. Prior to analysis, metabolite concentrations were log_2_-transformed and standardized to units of standard deviation. Multiple testing correction was applied using the Benjamini–Hochberg false discovery rate (FDR) method separately for each metabolomics platform, per biomarker, and within each stratified analysis (sex and APOE genotype).

To assess potential effect modification, we conducted stratified analyses by sex and APOE genotype. In the main analysis, linear regression models were run for all participants, with adjustment for covariates including APOE ε4 carrier status. To explore sex differences, we repeated the regressions separately for men and women, omitting sex as a covariate. Similarly, APOE-stratified models were conducted within genotype strata, where APOE was treated as an effect modifier rather than a confounder and therefore not included as a covariate. For the APOE analyses, participants were classified into three groups: ε2 carriers (ε2/ε2 or ε2/ε3), ε3 homozygotes (ε3/ε3, reference group), and ε4 carriers (ε3/ε4 or ε4/ε4). Individuals with the ε2/ε4 genotype were excluded to avoid opposing effects within the same group. In stratified models, APOE was omitted as a covariate. Differences between APOE ε2 and ε4 carriers were estimated by contrasting regression coefficients obtained from the stratified models. For each metabolite–biomarker association, we subtracted the ε4 effect estimate from the ε2 estimate and calculated a Wald-type z statistic using the combined standard errors from both strata. Two-sided p-values for the difference were derived from the standard normal distribution.

To minimize potential reverse causation and ensure that observed associations reflect early metabolic changes rather than consequences of advanced disease, we repeated all metabolite–biomarker association models after excluding participants with dementia or stroke at time of blood sampling. Additionally, we repeated the metabolite regressions after excluding participants with impaired kidney function (eGFR ≤ 60).

## Results

### Study population and metabolite profiling

**Fig. 1:**
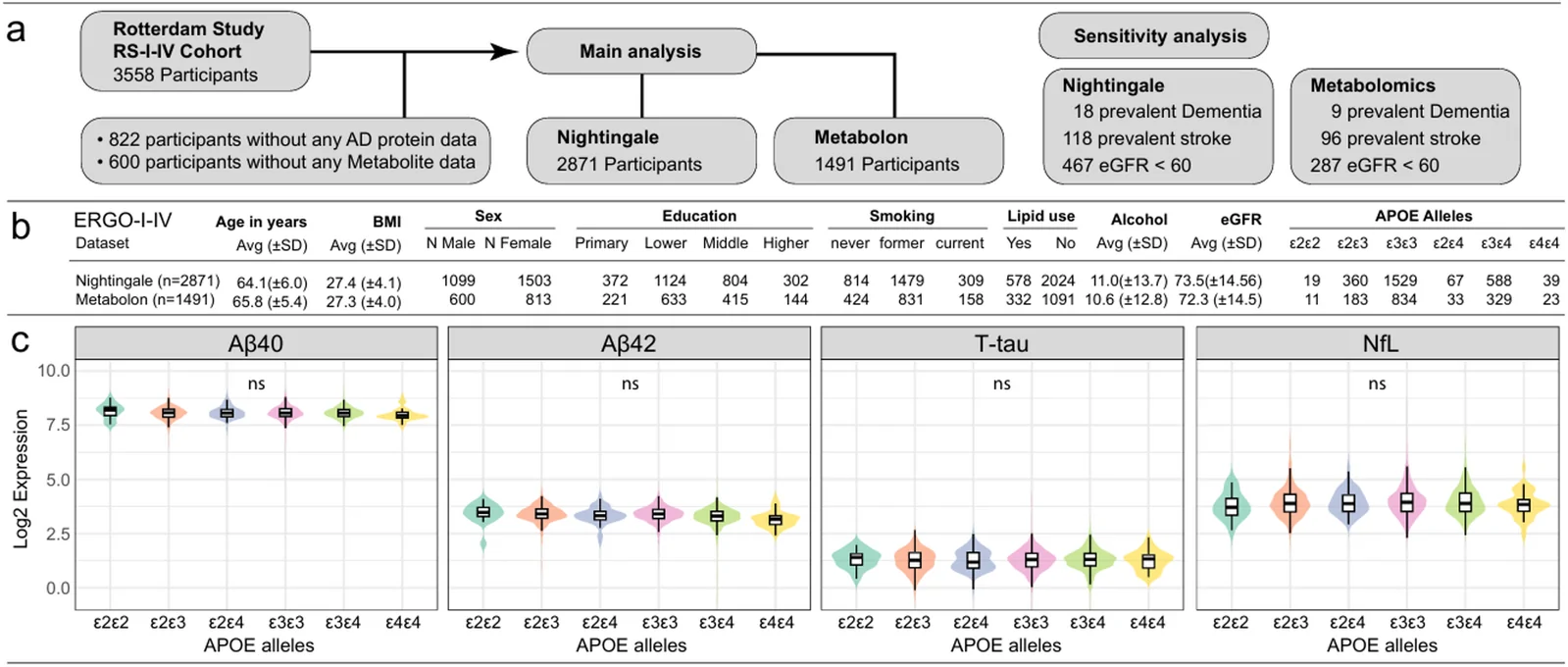
Overview of the Rotterdam Study (RS-I–IV) cohort and analytic datasets. **A** Flow of participants included in the analyses, showing exclusions and the resulting sample sizes for the Nightingale and Metabolon datasets. **B** Baseline characteristics of participants, including age, BMI, sex distribution, education, smoking status, lipid medication use, alcohol intake, kidney function (eGFR), and distribution of APOE genotypes. **C** Distribution of Alzheimer’s disease–related protein biomarkers (Aβ40, Aβ42, tau, and neurofilament light [NfL]) across APOE genotype groups, shown as violin plots with embedded boxplots of log2-transformed expression levels

A total of 2,958 participants from the Rotterdam Study were included in this analysis, with available measurements for Alzheimer’s disease protein biomarkers and metabolomic profiles. Figure 1A shows a flow diagram of participants included in different analyses. Figure 1B summarizes key characteristics of the study population, including age, sex, BMI, and APOE ε4 allele status for participants having data from the metabolomics platforms. A total of 2,871 participants with Nightingale metabolite profiling and 1,491 participants from the Metabolon metabolite profiling were included, with 1,404 participants having data from both, resulting in 2,958 unique participants. For Nightingale subset, the mean age was 64.1 years (SD 6.0), with 52.6% female participants, a mean BMI of 27.4 kg/m² (SD 4.1), and 19.4% APOE ε4 carriers. In the Metabolon subset, the mean age was 65.8 years (SD 5.4), with 59.7% female participants, a mean BMI of 27.3 kg/m² (SD 4.0), and 20.1% APOE ε4 carriers. The boxplots from Fig. 1C show the distribution of Alzheimer’s disease biomarker plasma concentrations across participants with different APOE alleles. No significant differences in plasma concentrations were observed between APOE alleles or between participants with the Nightingale and Metabolon data (Supplementary Figure S1).

### Associations between metabolites and Alzheimer’s disease protein biomarkers

**Fig. 2:**
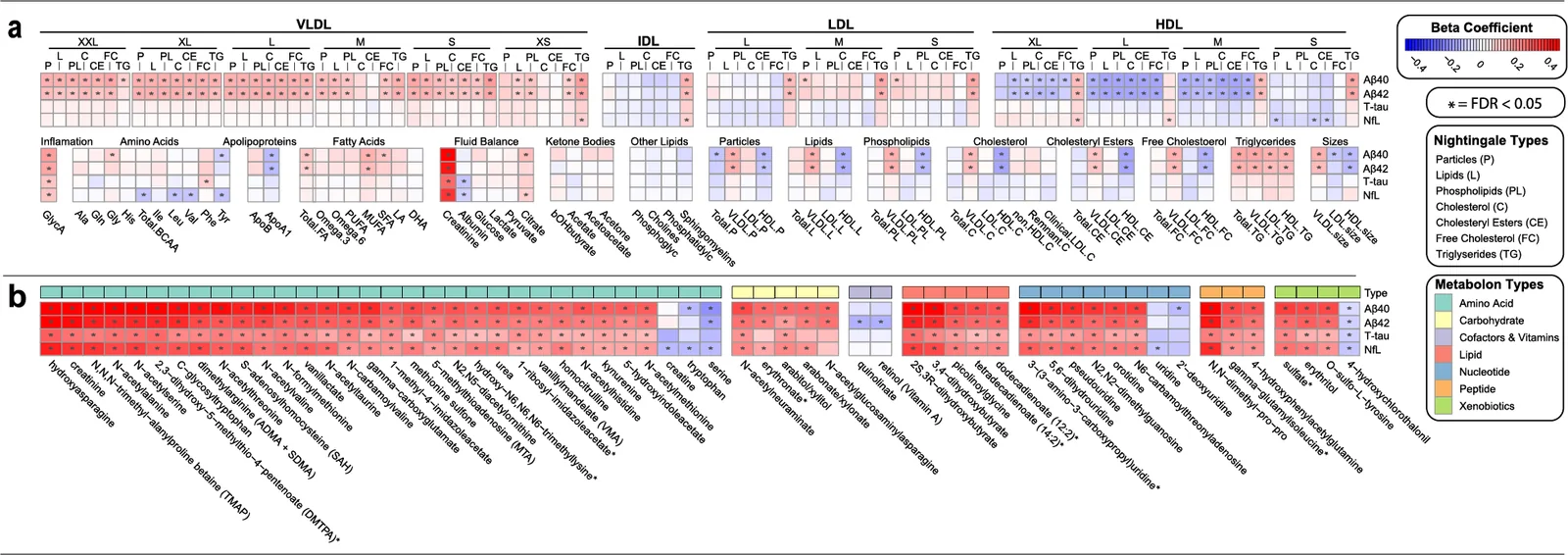
Associations between metabolites and Alzheimer’s disease–related protein biomarkers. **A** Heatmap of associations from the Nightingale dataset, grouped by lipoprotein subclasses, lipids, amino acids, apolipoproteins, fatty acids, fluid balance markers, ketone bodies, other lipids, and particle measures. Each square represents the regression coefficient for a metabolite–protein pair, with color indicating effect direction and magnitude (blue = negative, red = positive). **B** Heatmap of associations from the Metabolon dataset, grouped by metabolite classes including amino acids, carbohydrates, cofactors and vitamins, lipids, nucleotides, peptides, and xenobiotics. Asterisks (*) indicate associations that remain significant after false discovery rate (FDR) correction

The results of associations between plasma circulating metabolites and four protein biomarkers from linear regression models adjusted for covariates are visualized in a heatmap in Fig. 2.

On the Nightingale platform, which captures a wide range of lipids and lipoprotein subclasses (Fig. 2A), triglyceride-rich lipoproteins across VLDL, LDL, and HDL subclasses showed strong positive associations (FDR < 1e-10) with Aβ40 and Aβ42, and consistent negative associations (FDR < 1e-7) were shown for HDL cholesterol fractions. The strongest association was observed between Triglyceride-rich lipoproteins in small VLDL with Aβ40 (β = 0.168, FDR = 4.362e-16). Also, positive associations were found for all four proteins with creatinine (β > 0.253, FDR < 3.371e-24) and GlycA (β > 0.097, FDR < 2.092e-05). T-tau (β = -0.094, FDR = 1.924e-04) and NfL (β = -0.079, FDR = 4.210e-05) levels showed negative associations with albumin. Moreover, t-tau was positively associated with phenylalanine (β = 0.085, FDR = 0.001), and NfL exhibited additional associations with several triglycerides, tyrosine (β = -0.098, FDR = 5.627e-07), valine (β = -0.097, FDR = 3.868e-06), leucine (β = -0.105, FDR = 8.380e-07), and branched-chain amino acids (BCAAs) (β = -0.093, FDR = 1.664e-05), as well as reduced smaller HDL subclasses.

On the Metabolon platform, many positive and some negative associations were observed among metabolites from different metabolic pathways and Alzheimer’s disease protein biomarkers, several of which have been previously associated with the disease pathology [12]. The most significant positive and negative associations, where at least one protein biomarker per metabolite was significant after multiple testing correction (FDR), are displayed in Fig. 2B. Uridine exhibited a statistically significant inverse association with NfL (β = -0.102, FDR = 5.508e-05). On the other hand, 2’-deoxyuridine showed a stronger negative association with Aβ40 (β = -0.119, FDR = 3.638e-05). S-adenosylhomocysteine (SAH) was positively associated with all four Alzheimer’s disease biomarkers (β > 0.206, FDR < 1.303e-09). The full regression results for both platforms, covering both significant and non-significant associations, are available in Supplementary Table S1A-B.

### Stratified analysis

To examine whether associations between metabolites and Alzheimer’s disease protein biomarkers varied by genetic risk or sex, we conducted stratified analyses by APOE genotype and sex. Most results were consistent across strata (presented in Supplementary Table S1C-F), with the exception of the following.

**Fig. 3:**
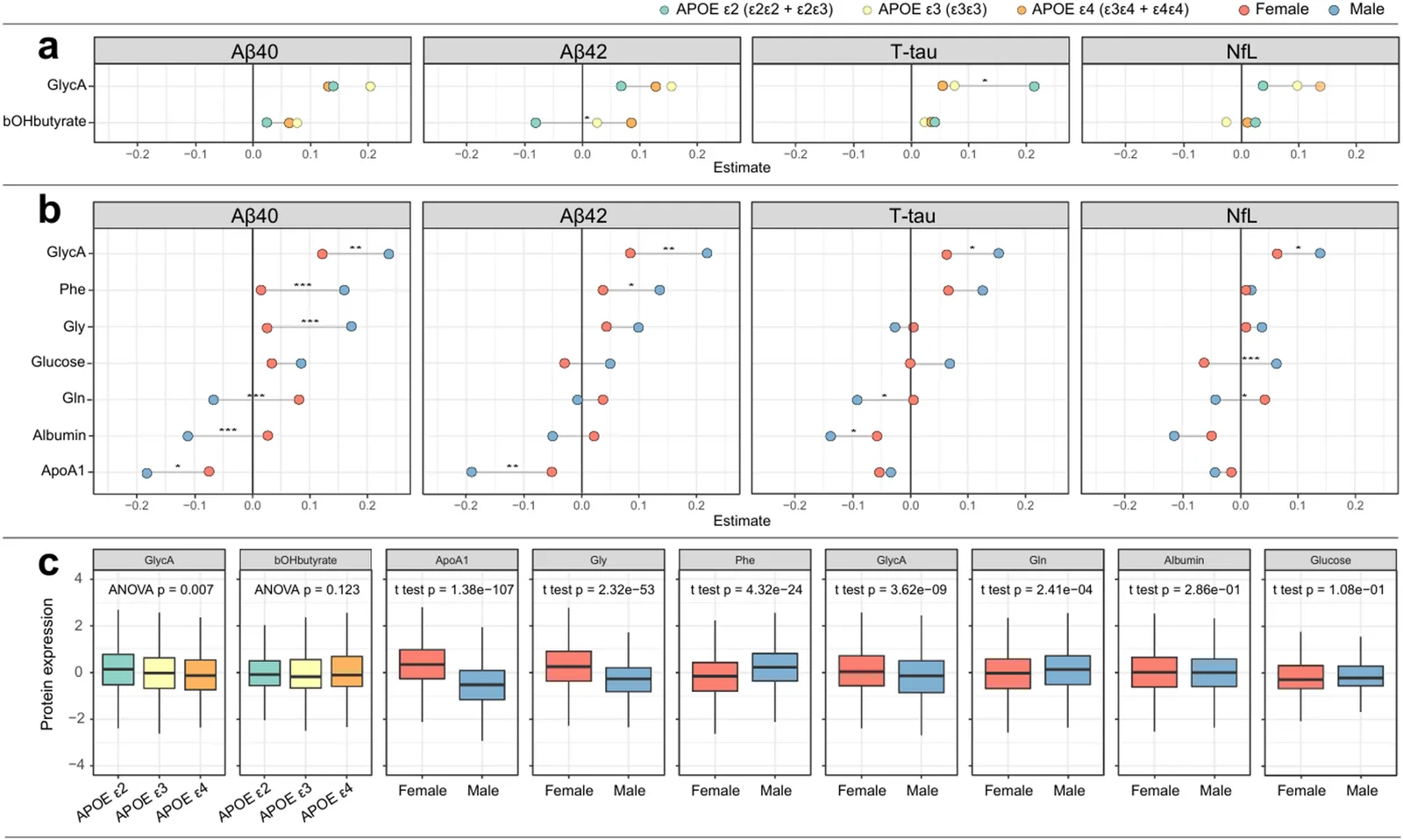
Associations of metabolites with Alzheimer’s disease–related protein biomarkers, stratified by APOE genotype and sex. Regression estimates of selected metabolites with Aβ40, Aβ42, tau, and neurofilament light (NfL), shown separately for APOE ε2 carriers (ε2ε2 + ε2ε3), ε3 carriers (ε3ε3), and APOE ε4 carriers (ε3ε4 + ε4ε4). **A** line is shown between the ε2 and ε4 dots and a star (*) indicates significant differences. **B** Regression estimates of selected metabolites with the same biomarkers, stratified by sex (female vs. male). Points represent effect estimates, with asterisks indicating statistical significance. **C** Boxplots of metabolite distributions across APOE genotype groups and sex with p values from tests shown for each panel

When stratifying by APOE genotype, we compared participants with the APOE ε2/ε2 or ε2/ε3 genotypes to those with APOE ε3/ε4 or ε4/ε4 genotypes in Fig. 3A. One of the largest differences was observed for the inflammatory marker GlycA in relation to t-tau: there was no significant association among APOE ε4 carriers, whereas a strong positive association (β = 0.216, FDR = 2.057e-04) was observed in APOE ε2 carriers. Although not statistically significant, β-hydroxybutyrate (bOHbutyrate) showed an APOE genotype-dependent trend in its association with plasma Aβ42 levels. A positive effect was observed in ε4 carriers, while a negative effect was seen in ε2 carriers. Additionally, VLDL particles showed trends toward stronger associations with Aβ40 in APOE ε4 carriers than in ε2 carriers, though these differences did not reach statistical significance.

Results from the sex stratified analysis with the largest differences between estimates is shown in Fig. 3B. The amino acids glycine and phenylalanine were more strongly associated with Aβ40 in males than in females. GlycA demonstrated robust positive associations with all four biomarkers in males, while the effect sizes were smaller or non-significant in females. Moreover, while albumin and glutamine (Gln) were negatively associated with Aβ40 in men, they were positively associated in women. Similarly, glucose was positively associated with NfL in men but negatively associated in women. Apolipoprotein A1 (ApoA1) showed strong negative associations with both Aβ42 and Aβ40 in men, whereas much weaker associations were observed in women. Finally, many nominally significant associations (*P* < 0.05) with HDL (positive direction) and VLDL (negative direction) lipoproteins were observed in women, but the associations were more robust in men, with larger effect sizes and surviving multiple testing correction. To further explore these findings, we examined group differences in baseline metabolite levels (Fig. 3C). GlycA levels differed across APOE strata (*p* = 0.007), while β-hydroxybutyrate did not. In sex-stratified comparisons, ApoA1, Gly, Phe, GlycA, Gln, and Albumin were significantly higher in one sex, whereas glucose showed no difference.

More differences by APOE genotype and sex were observed in our analysis with diverse metabolites in the Metabolon platform, as shown in Supplementary Figure S2. Of note, significant associations were observed with specific metabolites among men such as bilirubin (E, E), a heme catabolite with reported antioxidant and neuroprotective functions in Alzheimer’s disease [13, 14], and quinolinate, a kynurenine pathway metabolite implicated in excitotoxicity and neuroinflammation [15, 16]. All stratified results from both metabolomics platforms and four Alzheimer’s disease biomarkers are available in Supplementary Table S1C-F.

### Sensitivity analysis

Excluding participants with prevalent dementia or stroke had negligible impact on the association results. Among participants with available Nightingale data (*n* = 2,871), 20 had prevalent dementia and 118 had a history of stroke, while in the Metabolon dataset (*n* = 1,491), 9 participants had prevalent dementia and 96 had a history of stroke. Effect estimates between the primary and sensitivity analyses were nearly identical (median r of betas ≥ 0.99 across biomarkers, Supplementary Table S2), with mean absolute differences < 0.01 and > 95% of associations retaining the same direction. We performed a second sensitivity analysis excluding participants with eGFR ≤ 60 (*n* = 467 for Nightingale and *n* = 287 for Metabolon), which also remained highly consistent with the main analyses. Across biomarkers and platforms, correlation coefficients for effect estimates ranged from 0.976 to 0.996, with mean absolute differences in beta values < 0.02 (Supplementary Table S3). Notably, creatinine remained significantly associated with all four Alzheimer’s disease biomarkers (Aβ40, Aβ42, t-tau, and NfL), in both Nightingale and Metabolon, in participants with preserved kidney function (eGFR > 60).

## Discussion

In this population-based study, we examined the relationships between plasma circulating metabolites and four core Alzheimer’s disease protein biomarkers (Aβ40, Aβ42, t-tau, and NfL), stratified by sex and APOE genotype, in nearly 3,000 participants. By combining targeted (Nightingale) and untargeted (Metabolon) metabolomics, we captured a broad spectrum of over 1000 metabolic signatures. This dual-platform approach enabled the identification of a range of lipid and non-lipid metabolites associated with the Alzheimer’s disease pathological hallmark proteins, while also revealing distinct metabolic phenotypes linked to APOE ε2 and ε4 carriers. These results offer insights into early systemic changes that may precede or parallel Alzheimer’s disease pathology and highlight stratified metabolic profiles.

Our findings suggest that lipoprotein particle size may be associated with amyloid regulation, with positive associations observed for VLDL size and negative associations for LDL and HDL sizes. Consistent with this, prior research has shown that larger LDL particles are linked to greater cerebral amyloid deposition and reduced hippocampal volume in cognitively unimpaired older adults, independent of total cholesterol levels [17]. In contrast, smaller HDL particles may be involved in Aβ clearance and help preserve brain structure, as they are more efficient at crossing the blood-brain barrier and interacting with key transport proteins [18].

We found that higher levels of triglyceride-rich lipoproteins were linked to increased plasma Aβ40 and Aβ42 levels. This aligns with previous studies showing that elevated triglycerides and reduced HDL cholesterol may impair Aβ clearance from the bloodstream [19].

However, other mechanisms may also contribute. Insulin resistance has been shown to disrupt insulin-degrading enzyme activity and peripheral Aβ metabolism, which may contribute to higher circulating Aβ levels [20, 21]. In addition, vascular dysfunction related to obesity and dyslipidemia has been linked to elevated plasma Aβ and may impair clearance at the neurovascular unit [22, 23]. At the same time, plasma Aβ levels reflect both central and peripheral processes and may not directly represent brain amyloid pathology. The observed associations may therefore also relate to differences in peripheral Aβ production, systemic clearance, including renal function, or measurement-related factors, and their direction remains uncertain. Thus, altered lipid levels may not only be associated with Aβ transport but may also reflect broader metabolic and vascular disturbances linked to Alzheimer’s disease pathology.

Most of the metabolites we identified were related to lipids or lipid pathways. In the brain, cholesterol metabolism is mostly independent from the circulation, but microglia rely heavily on lipid handling to clear or form amyloid plaques [24]. At the same time, lipids and lipoproteins in the blood can influence how Aβ moves across the blood–brain barrier, affecting its balance between brain and circulation [25]. These findings suggest that lipid changes in the blood may reflect peripheral metabolism and may also contribute to amyloid accumulation in the brain.

NfL and tau, known as markers of neuroaxonal damage and neurofibrillary pathology respectively, showed distinct patterns of association with circulating metabolites in this study. NfL was negatively associated with several amino acids, including tyrosine, valine, leucine, and other branched-chain amino acids (BCAAs), as well as with smaller HDL subclasses. Prior studies have reported associations between circulating BCAAs and both Alzheimer’s disease risk and cognitive impairment, supporting the role of altered amino acid metabolism in early neurodegeneration [26, 27]. Mendelian Randomization evidence further supports a potential causal link, particularly for isoleucine, which may increase Alzheimer’s disease risk by competing with tryptophan and tyrosine at the blood-brain barrier [28]. Such competition may reduce brain uptake of these amino acids, thereby limiting availability of neurotransmitter precursors like serotonin and dopamine [29, 30].

Furthermore, creatinine was positively associated with all four protein biomarkers in our study, despite adjusting for eGFR and excluding participants with eGFR < 60. Creatinine is a marker of systemic renal clearance, and thus these may reflect altered systemic clearance of Alzheimer’s disease-related proteins from the bloodstream rather than from the brain itself. However, given that eGFR is partly derived from creatinine, the inclusion of both measures may introduce collinearity, and residual confounding by kidney function cannot be fully excluded. Previous work has shown that lower eGFR is associated with higher concentrations of Aβ40, Aβ42, NfL, and GFAP [31]. Elevated GlycA, which was also consistently associated with all four biomarkers, has previously been linked to reduced brain volumes and cognitive decline in Alzheimer disease, suggesting that systemic inflammation may contribute to neurodegeneration [32].

We further observed elevated S-adenosylhomocysteine levels across all Alzheimer’s disease protein biomarkers. S-adenosylhomocysteine is a product of methylation reactions and a potent inhibitor of methyltransferases. Its accumulation suggests impaired one-carbon metabolism and reduced methylation capacity, which may compromise DNA, protein, and phospholipid methylation. Such epigenetic and metabolic dysregulation has been implicated in tau hyperphosphorylation and increased neuronal vulnerability [12, 33, 34].

Overall, the APOE-stratified analyses showed largely similar metabolite–biomarker associations across ε2 and ε4 carriers, suggesting that most associations are not strongly modified by APOE genotype. Nevertheless, a few differences were notable: GlycA showed stronger positive associations with t-tau in ε2 carriers compared to ε4 carriers, indicating a possible genotype-specific link between systemic inflammation and tau pathology. In addition, β-hydroxybutyrate demonstrated opposing associations with Aβ42 across APOE strata, hinting at potential interactions between ketone metabolism and APOE-related mechanisms of amyloid processing.

Our sex-stratified analyses revealed more metabolite associations in men than in women, particularly for Aβ40 and Aβ42, despite a slightly smaller male subgroup. For instance, GlycA, phenylalanine, and several amino acids showed robust associations with Alzheimer’s disease biomarkers in males, while the corresponding associations were weaker or absent in females. Similarly, apolipoprotein A1 (ApoA1) was negatively associated with Aβ40 and Aβ42 in men only, suggesting a potential sex-specific role of lipid transport in amyloid metabolism. Female APOE ε4 carriers show stronger amyloid and tau pathology [35, 36], potentially linked to hormone-sensitive energy metabolism [37, 38], whereas our findings in men suggest a comparatively more inflammation- and lipid-driven profile.

We further examined the stratified results by looking at the baseline differences, which are important because they may contribute to the apparent heterogeneity of metabolite–biomarker associations in stratified models. For example, higher GlycA levels across APOE genotypes or sex could partly explain variations in effect estimates. In contrast, β-hydroxybutyrate showed no baseline differences across APOE groups, suggesting that the divergent associations observed reflect genuine APOE-specific modification rather than underlying expression shifts. This interpretation is further supported by large population-based evidence showing that energy metabolism–related metabolites, such as β-hydroxybutyrate, are influenced by age and APOE genotype and are differentially associated with the disease risk [39].

Our sensitivity analysis excluding individuals with prevalent Alzheimer’s disease or stroke confirmed that the observed associations between blood metabolites and Alzheimer’s disease biomarkers were not driven by existing neurodegenerative or cerebrovascular disease at baseline.

The key strengths of our study include the use of both targeted NMR- and untargeted MS-based metabolomic platforms. This dual approach enhances coverage and specificity, capturing numerous metabolic features relevant to AD. Importantly, the metabolome complements the signature of disease, offering critical insights into the biological processes underlying AD. By linking metabolite data with protein markers, which are becoming increasingly central to AD diagnosis and progression tracking, we demonstrate how metabolomics can provide additional layers of understanding that strengthen the utility of protein biomarkers. Together, these approaches create a more comprehensive and powerful framework for identifying biomarkers and understanding AD pathophysiology. Additionally, the use of a large, well-characterized population-based cohort with a stratified design allows us to uncover subgroup-specific signals, enhancing the ability to detect nuanced differences in metabolic responses. This provides better insights into the heterogeneity of AD, particularly in relation to genetic and biological sex influences. Our results confirm and expand on previous AD cohort findings, highlighting the potential of combining metabolomic and protein markers for more accurate disease profiling and interventions.

Several limitations of our study should also be acknowledged. First, the cross-sectional design prevents the ability to infer causality or temporal relationships between metabolite levels and Alzheimer’s disease biomarkers. Second, statistical power was reduced in stratified models, which may have limited the detection of subtler associations. Third, genotype-specific comparisons may not generalize to excluded individuals with the APOE ε2/ε4 genotype. Fourth, while our combined NMR and MS platforms provide broad metabolic coverage, certain biologically relevant metabolite classes (e.g., neurotransmitters, redox molecules) are not fully captured by current platforms. Fifth, although total tau was included in our analyses, phosphorylated tau (p-tau), a more specific and accurate biomarker of Alzheimer’s disease pathology, was not available in our cohort, which may limit the precision of our findings related to tau pathology. Finally, comparison of two plasma markers could be confounded by factors that influence binding or clearance of both markers, if incompletely adjusted for in the analyses.

## Conclusions

Our findings demonstrate that metabolic alterations in lipids, inflammation, and amino acid metabolism are closely linked to Alzheimer’s disease pathological processes. The stratified analyses suggest that these systemic pathways may contribute differently across subgroups, with sex-specific patterns pointing to distinct biological vulnerabilities. Our results highlight potential mechanistic links between peripheral metabolism and core Alzheimer’s disease protein changes, including impaired lipid transport, systemic inflammation, and disrupted amino acid and methylation pathways. These findings contribute to a broader understanding of the disease, indicating that systemic metabolic disturbances may precede or interact with brain pathology. Future longitudinal and multi-omic studies are warranted to clarify causality and disentangle how these peripheral processes influence neurodegeneration; additionally, age-stratified analyses may reveal heterogeneity in metabolite-biomarker associations.

## Supplementary Information

Supplementary Material 1: Figures S1 and S2.

Supplementary Material 2: Supplementary Table 1. Output of all statistical tests.

Supplementary Material 3: Supplementary Table 2. Output of sensitivity analysis removing prevalent Dementia and Stroke.

Supplementary Material 4: Supplementary Table 3. Output of sensitivity analysis removing EGFR <= 60.

## Data Availability

The Rotterdam Study data can be made available to interested researchers upon request. Requests can be directed to data manager Frank J. A. van Rooij (f.vanrooij@erasmusmc.nl) or visit the following website for more information: https://www.ergo-onderzoek.nl/contact. We are unable to place data in a public repository due to the confidential nature of the data collected and legal and ethical constraints, but the full summary statistics of our results can be released through https://www.mirnomics.com/.

## Abbreviations

Aβ: Amyloid-β
NfL: Neurofilament light chain
T-tau: Total tau
FDA: Food and Drug Administration
APOE: Apolipoprotein E
NMR: Nuclear magnetic resonance
BMI: Body mass index
eGFR: Estimated glomerular filtration rate
FDR: False discovery rate
BCAAs: Branched-chain amino acids
SAH: S-adenosylhomocysteine
bOHbutyrate: β-hydroxybutyrate
Gln: Glutamine
ApoA1: Apolipoprotein A1
