# Supplementary material for: Associations of plasma metabolites with protein biomarkers linked to Alzheimer’s disease pathology in the Rotterdam Study
Source: Alzheimers Res Ther. 2026 Jun 10;18:225. doi: 10.1186/s13195-026-02108-2 (PMC13647584; doi:10.1186/s13195-026-02108-2)
Supplement: Supplementary file 1 [file 13195_2026_2108_MOESM1_ESM.docx]

Figure S1: The figure illustrates the log-transformed normalized expression levels of four biomarkers (Tau, NFL, Aβ40, and Aβ42) across two metabolomics platforms (Nightingale and Metabolon) in relation to different confidence interval (CI) levels. The first panel on the left shows the distribution of log-normalized expression with CI levels represented by distinct colors. The middle and right panels depict boxplots of the log2-transformed expression values for each biomarker, organized by platform and stratified by APOE subtype. This presentation highlights the variation in biomarker expression across the different CI levels, platforms, and biomarkers.

Figure S2: The figure displays estimates of metabolite associations across different subgroups. The left panel shows sex-stratified associations, with estimates for females (red) and males (blue) for each metabolite. The middle panel presents associations stratified by APOE ε2 carrier status, with carriers and non-carriers indicated by green and yellow, respectively. Statistical significance is indicated by asterisks next to each estimate, providing insight into how these metabolites are influenced by sex and APOE genotype. The figure highlights significant differences in metabolite levels across these strata, suggesting potential interactions with sex and genetic background.
